# Ethics and methods in disaster health research: a scoping review

**DOI:** 10.7189/jogh.16.04235

**Published:** 2026-06-19

**Authors:** Krzysztof Goniewicz, Amir Khorram-Manesh, Katarzyna Naylor, Eric Carlström, Axel Wolf

**Affiliations:** 1 Polish Air Force University, Deblin, Poland; 2 Department of Surgery, Institute of Clinical Sciences, Sahlgrenska Academy, University of Gothenburg, Gothenburg, Sweden; 3 Centre for Disaster Medicine, University of Gothenburg, Gothenburg, Sweden; 4 Gothenburg Emergency Medicine Research Group, Sahlgrenska University Hospital, Gothenburg, Sweden; 5 Independent Unit of Emergency Medical Services and Specialist Emergency, Medical University of Lublin, Lublin, Poland; 6 Institute of Health Care Sciences, University of Gothenburg, Gothenburg, Sweden; 7 University of Gothenburg Centre for Person-Centred Care (GPCC), Sahlgrenska Academy, University of Gothenburg, Sweden; 8 Institute of Nursing and Health Promotion, Oslo Metropolitan University, Oslo, Norway; 9 Department of Anaesthesiology, Intensive Care Medicine and Pain Medicine, Region Västra Götaland, Sahlgrenska University Hospital, Gothenburg, Sweden

## Abstract

**Background:**

Disaster medicine operates in unstable, high-risk environments where operational, ethical, and contextual constraints frequently undermine traditional research designs. Although global health agendas increasingly emphasise person- and patient-centred care (PCC), these principles remain poorly articulated and rarely operationalised within disaster research. We aimed to examine how ethical tensions and methodological limitations shape the current disaster health research evidence base and explore how PCC might be meaningfully integrated into research design and governance.

**Methods:**

We conducted a structured scoping review of peer-reviewed literature across PubMed, Scopus, and Web of Science, focusing on research methodology, ethics, and evidence generation in disaster and humanitarian contexts. We thematically synthesised the extracted data.

**Results:**

We included 28 studies, identifying three cross-cutting thematic domains: ethical tensions related to participant vulnerability and governance in crisis settings; methodological constraints limiting the generation of robust, context-sensitive evidence; and inconsistent application of PCC principles.

**Conclusions:**

The evidence base for disaster medicine appears conceptually rich but structurally fragile. Ethical and methodological constraints suggest a critical gap in the operationalisation of PCC within disaster research. Advancing the field will likely require adaptive methodologies, context-responsive ethical oversight, and more explicit integration of person-centred principles into research design and evaluation, particularly in protracted crises and recovery settings where resilient systems must integrate health and social care.

**Registration:**

Open Science Framework: https://doi.org/10.17605/OSF.IO/3A8CZ.

Disaster medicine occupies a uniquely challenging intersection of science, ethics, and emergency response. Unlike conventional clinical environments, disasters unfold in unstable, unpredictable, and resource-constrained settings where acute threats to life routinely disrupt the established structures, timelines, and governance mechanisms that typically support health research [1]. Earthquakes, conflict, forced displacement, and large-scale outbreaks increasingly shape global health realities, yet the evidence base guiding medical decision-making in these environments remains fragmented and inconsistent [2]. The consequences are immediate: clinicians and responders must act decisively while facing profound uncertainty, often without the methodological support that typically underpins evidence-based care. In this review, disasters are understood as events that severely disrupt the functioning of communities and health systems, including natural hazards, armed conflicts, epidemics, and humanitarian crises.

Nevertheless, many contemporary crises are not discrete or time-limited events, but rather prolonged and recurrent disruptions, including armed conflicts, protracted displacement, and repeated outbreak surges, that reshape the lives of individuals, families, and communities over months or years, both during active crisis phases (*e.g.* war or large-scale natural catastrophes) and throughout extended periods of recovery. In such contexts, health outcomes depend not only on acute clinical interventions but also on continuity of care, trust, communication, and sustained social support. This reality underscores the need for disaster response systems that are resilient across temporal phases and institutional sectors, integrating person-centred healthcare with social care and community-based resources.

Accordingly, person-centred systems must operate coherently across multiple, interdependent levels. At the micro level (*i.e.* clinical encounter), this involves attention to dignity, meaningful communication, respect for individual values and preferences, and shared decision-making, even under severe constraints. At the meso level (*i.e.* service and organisational pathways), person-centredness depends on continuity and coordination of care, family support, culturally safe interfaces, and effective transitions across care settings, such as between emergency departments, shelters, primary care, and rehabilitation services. At the macro level (*i.e.* systems and governance), it requires participation, accountability, and equity, alongside integration of health and social care, responsible data governance, and adequate ethical oversight capacity [3]. Within this perspective, person-centred care (PCC) should be understood not merely as a normative aspiration, but as an integrating ethical stance, organising logic, and evidence-informed methodological approach across both health and social care during crisis response and recovery.

We selected PCC as an interpretive framework because it provides an operational bridge between ethical principles and practical research conduct, explicitly articulating constructs such as dignity, autonomy, participation, and responsiveness to lived experience. While alternative ethical frameworks offer important normative guidance, we consider PCC especially useful for examining how these principles are translated into relational and methodological practices within disaster research.

Here, we did not treat PCC as the primary subject of analysis but rather as an interpretive framework for examining how ethical and relational dimensions of disaster research are addressed in the literature. Evidence from person-centred clinical research suggests positive medical, social, organisational, and economic outcomes when compared with conventional care models [4,5].

Yet, despite this imperative and evidence, the literature reveals a striking gap: principles consistent with PCC are seldom explicitly articulated, operationalised, or evaluated within disaster medicine, leaving both ethical practice and methodological rigour inadequately supported [6,7].

These tensions raise a set of fundamental questions for the field: why do conventional research designs face limitations in disaster contexts, and how do such failures expose limitations in established ethical frameworks? How can researchers generate credible evidence under conditions that disrupt normal study design, informed consent procedures, and participant protection mechanisms? And, crucially, what methodological innovations or adaptive research paradigms exist that could align the realities of disaster settings with the ethical and relational commitments inherent in PCC?

Addressing these questions is essential not only for improving scientific rigour but also for safeguarding the dignity and agency of individuals affected by crises. As disasters escalate in frequency and scale worldwide, the need for a clearer conceptual and methodological foundation becomes urgent. We sought to respond to that need by examining the constraints that undermine traditional research approaches, analysing the ethical tensions created by rapid decision-making in unstable environments, and synthesising emerging adaptive methodologies that may better support both rigorous evidence generation and person-centred practice. We aimed to examine how ethical and methodological challenges in disaster-related health research are conceptualised in the literature and to explore the extent to which these discussions engage with principles consistent with PCC.

## METHODS

### Study design

We employed a structured scoping review design, following the methodological guidance of Arksey and O’Malley [8], refined by Levac *et al.* [9], and reported in accordance with the PRISMA-ScR framework [10]. We prospectively developed the review protocol and registered it in the Open Science Framework, providing procedural transparency and ensuring methodological fidelity across all stages of the review process.

Given the inherent methodological and ethical constraints of disaster medicine, characterised by unpredictable environments, acute humanitarian needs, and contextual variability, this design enabled a comprehensive mapping of methodological, ethical, and person-centred considerations in disaster research. A scoping approach was therefore most appropriate for capturing the breadth of conceptual contributions, methodological adaptations, and ethical imperatives documented across diverse empirical and theoretical sources.

We additionally adopted a person-centred analytic lens inspired by the Gothenburg Framework for Person-Centred Care [5], which we selected due to its explicit operationalisation of relational and ethical dimensions of care. This approach recognises that research conducted in disaster environments is inextricably intertwined with issues of dignity, autonomy, vulnerability, and relational ethics. Accordingly, we constructed the methodological framework to identify not only technical study-design challenges but also the PCC/person-centred elements (PCE) embedded within or absent from existing approaches.

The review team consisted of two primary reviewers (AKM and KG), who jointly executed all stages of screening and extraction, and a third senior reviewer (AW), who served as methodological adjudicator in cases of uncertainty or disagreement. This three-step review structure ensured both analytic consistency and interpretative rigour across the review’s iterative processes.

Our review was guided by the following research question: ‘How are ethical and methodological challenges in disaster-related health research conceptualised in the literature, and to what extent are these discussions aligned with principles consistent with PCC?’.

### Search strategy

We conducted a comprehensive search across PubMed, Scopus, and Web of Science. The strategy combined controlled vocabulary and free-text terms related to disaster medicine, mass casualty incidents, humanitarian crises, research ethics, study design, evidence generation, and PCC. We employed the Boolean operators (*i.e.* AND, OR, and NOT) to ensure conceptual breadth while maintaining relevance to the methodological and ethical dimensions of disaster-related research. The search strategy intentionally combined disaster-related terms with concepts related to research methodology, ethics, and PCC. This was done to capture literature on ethical and methodological challenges in disaster research, rather than purely clinical or operational studies. We iteratively developed the search strategy to balance conceptual relevance with specificity to the review question. However, we recognise that relevant literature may also be indexed under adjacent terminology such as humanitarian emergencies, crisis research, implementation science, participatory methods, community engagement, trauma-informed care, resilience, or health systems governance. The final strategy may therefore have favoured conceptual specificity over maximal sensitivity, and this should be considered when interpreting the scope of the included evidence base.

We conducted a comprehensive search across PubMed, Scopus, and Web of Science. The search strategy was designed to identify literature addressing three interconnected domains: disaster and humanitarian contexts, including disaster medicine, mass-casualty incidents, catastrophes, and humanitarian crises; research methodology and evidence generation, including study design, methodological approaches, and evidence-based practice; and ethical and person-centred dimensions of health research, including research ethics, dignity, respect, patient-centred care, and person-centred care. Search terms were combined using Boolean operators and adapted where necessary to the indexing requirements of each database to ensure both conceptual breadth and relevance to the review question.

We limited the searches to peer-reviewed articles and reviews published in English. We did not impose restrictions on publication date to ensure comprehensive historical coverage of methodological developments in the field. We also employed hand-searching of reference lists and citation tracking, further minimising the risk of omission. Despite the absence of date restrictions, most included studies were published within the last decade; this pattern may reflect changes in database indexing, shifts in terminology, and the comparatively recent consolidation of disaster research ethics and methodology as a more explicitly defined field of inquiry.

### Eligibility criteria

We developed the eligibility criteria *a priori*, which were aligned with the study’s conceptual focus on methodology and ethics in disaster-related health research. We included empirical articles, reviews, conceptual analyses, consensus statements, or methodological reports; studies that addressed research design, ethics, data collection, or evidence generation in disaster or humanitarian contexts; articles published in English; and studies that explicitly focused on human health or medical response. We excluded articles that were purely epidemiological or anecdotal descriptions of single disasters lacking methodological or ethical analysis; studies that focused on veterinary/animal health; and studies that included engineering, economics, or logistical modelling without a medical research component.

We included consensus statements, methodological reports, and expert-driven analyses only when published in peer-reviewed journals and when they made a substantive contribution to research ethics, research design, or evidence-generation challenges in disaster-related health contexts. We did not include grey literature, non-peer-reviewed reports, and opinion pieces without analytic, methodological or ethical content.

### Study selection

We imported all records identified through the searches into a reference management system and removed duplicates. Screening was conducted in two stages: two independent reviewers (AKM and KG) first screened titles and abstracts, and then did the full-text review applying the eligibility criteria afterwards.

Disagreements at any stage were discussed between the two primary reviewers (AKM and KG). When consensus could not be achieved, the third reviewer (AW) acted as an adjudicator, providing methodological oversight and ensuring alignment with the review protocol.

The searches yielded 239 records. After removing 76 duplicates, we screened 163 unique records by title and abstract. Of these, we excluded 83 primarily because they focused on clinical management, operational response, epidemiological description, or disaster preparedness without substantive engagement with research methodology, evidence generation, or ethical analysis. We screened 80 full-text articles for retrieval; two could not be assessed further because one full-text was unavailable, and one article was published in German and therefore fell outside the language criteria.

Therefore, we assessed 78 full-text articles for eligibility and excluded 50 because they did not substantively address methodological issues, ethical analysis, or human health research in disaster or humanitarian contexts. In total, 28 studies met all eligibility criteria and were included in the final synthesis.

**Figure 1:**
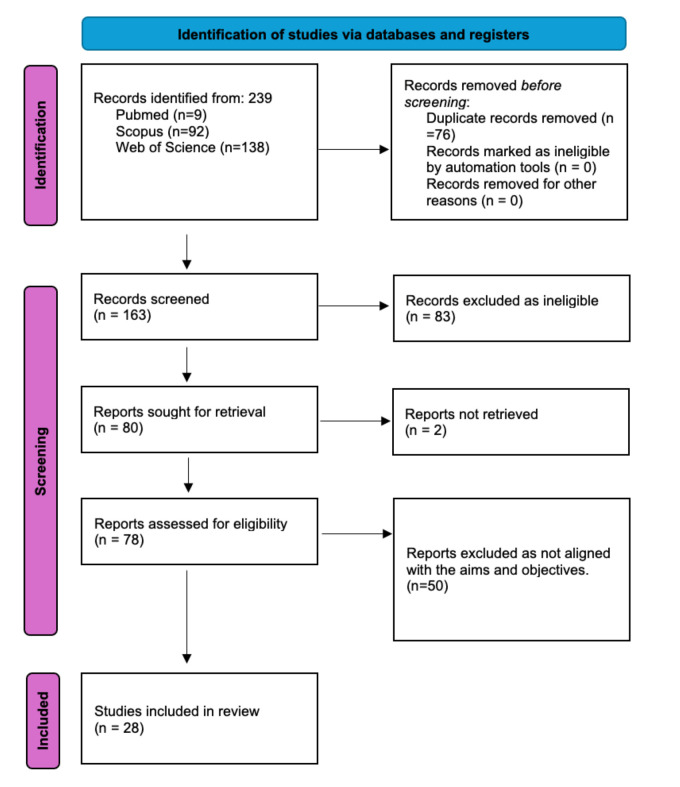
PRISMA-ScR flowchart [10].

Initial agreement between reviewers was generally high; however, three records were designated as ‘uncertain’, and 14 screening conflicts remained after the initial independent assessment and were subsequently resolved through discussion and, where necessary, adjudication by the third reviewer (**Figure 1**). Although we did not calculate formal inter-rater agreement statistics (*e.g.* Cohen’s kappa), we ensured procedural consistency through independent duplicate screening, structured discussion of disagreements, and third-reviewer adjudication for unresolved cases.

We conducted all screening and deduplication procedures using Rayyan (Qatar Computing Research Institute), which enabled blinded independent review, automatic duplicate detection, and structured resolution of conflicts.

### Data extraction

Two reviewers (AKM and KG) independently applied a structured data extraction form, developed iteratively and piloted on a subset of included studies. Extracted data covered two complementary domains: study characteristics (*e.g.* publication details, disaster context, study design, and primary focus) and analytic dimensions (*e.g.* ethical issues, methodological challenges, and the presence or absence of PCC elements).

The reviewers resolved any discrepancies in data extraction or coding through discussion, with the senior reviewer (AW) resolving all unresolved cases, ensuring analytic consistency and minimising interpretative bias.

### Data synthesis

Given the diversity of study designs and conceptual contributions, we employed a qualitative thematic synthesis. The two reviewers (AKM and KG) generated the initial codes inductively from the data during repeated reading of the included studies, focusing on segments related to ethical issues, methodological challenges, and PCE. We conducted the coding manually without the use of specialised qualitative analysis software. The two reviewers independently coded the initial subset of the included studies to refine the coding structure and improve consistency in interpretation. We compared, discussed, and merged the codes into higher-order categories through iterative team discussion. We reached the agreement for the final thematic domains only after the review team judged that they were analytically coherent, sufficiently distinct from one another, and adequately grounded in the included studies.

We descriptively used quantitative characterisation (*e.g.* frequency of study types and methodological approaches) to contextualise thematic findings. We synthesised cross-cutting themes to generate an integrative framework outlining recurrent methodological, ethical, and person-centred considerations relevant to future disaster medicine research.

To address potential reflexivity and minimise interpretive bias, particularly in relation to the inclusion of prior work, we conducted the coding and theme development collaboratively, with disagreements resolved through discussion and adjudication. The analytic process emphasised transparency and consistency across all included studies, regardless of authorship. No study was given privileged analytic weight based on authorship.

### Quality appraisal

In line with standard scoping review methodology [11], we did not conduct a formal critical appraisal of study quality. We aimed to map the breadth and conceptual characteristics of the literature rather than to evaluate methodological quality or risk of bias.

## RESULTS

A total of 28 studies met the eligibility criteria and were included in the final synthesis. The studies varied substantially in scope, design, disciplinary orientation, and crisis context, reflecting the conceptual and methodological heterogeneity characteristic of disaster medicine research. Included publications encompassed qualitative interviews, systematic and scoping reviews, expert consensus statements, policy analyses, and conceptual or reflective contributions. Collectively, they spanned a wide range of settings, including natural hazards, infectious disease outbreaks, humanitarian crises, protracted conflicts, and long-term recovery environments.

**Figure 2:**
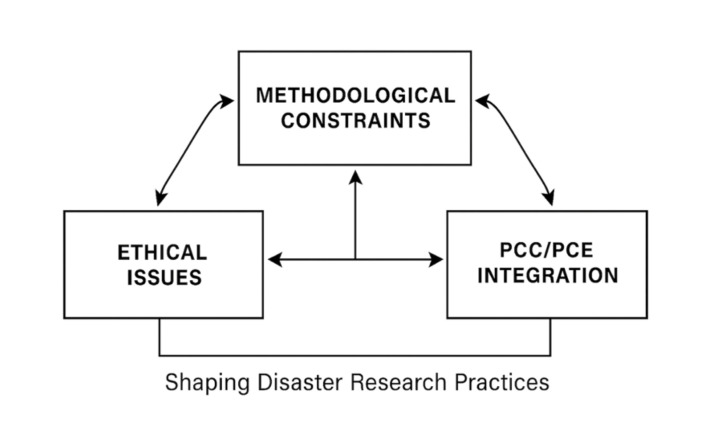
Conceptual model of the three thematic domains shaping disaster research practices.

Synthesis of the full-text material revealed three overarching thematic domains, which together structure how disaster research is conceptualised and operationalised (**Figure 2**). These domains reflect the types of ethical tensions that arise when conducting research in unstable or high-risk environments, the methodological limitations and structural constraints affecting the generation of reliable and contextually appropriate evidence, and how principles of person-centred or patient-centred care are integrated, adapted, or neglected within disaster research practices.

These domains provide the analytic foundation for interpreting the characteristics of the included studies and guide the detailed thematic synthesis presented in the sections that follow. Because the search strategy and review question explicitly targeted ethics, methodology, and PCC, these domains should be understood as partly emergent from the included literature and partly shaped by the conceptual framing of the review. They therefore represent an interpretive synthesis rather than purely inductive discovery.

### Characteristics of included studies

Most studies were conceptual analyses, reflective commentaries, or expert-driven consensus reports published in peer-reviewed journals, while fewer employed empirical methodologies such as qualitative interviews, systematic reviews, or mixed-methods approach (**Table 1**). Several papers operated at the interface of ethics, public health policy, and emergency management, highlighting the cross-sectoral nature of disaster research.

**Table 1:** Characteristics of the included studies (n = 28)

Study, year, reference	Type of disaster/context	Study type/design	Primary aim/focus
Aarons (2018) [12]	Epidemics and emergencies (Caribbean region)	Qualitative study (key informant interviews and literature-based framework development)	Develop a model for collaboration and expedited ethics review across RECs during epidemics/emergencies
Aarons (2019) [13]	Disasters and disease outbreaks	Conceptual critique informed by interviews/ethics review model	Assess the feasibility of CIOMS Guideline 20 and propose an ad hoc REC model for expedited ethical review in emergencies
Abeysinghe and Leppold (2023) [14]	Health and disaster research ethics (various disaster contexts)	Scoping review and qualitative frame analysis	Identify dominant framings of ethical practice and propose directions for improving ethical research in disaster settings
Afifi *et al.* (2020) [15]	Humanitarian crises; displaced and refugee communities	Conceptual paper with multiple case studies	Examine the application of CBPR principles in humanitarian crises and show how CBPR can recalibrate equity and power in vulnerable contexts
Aung *et al.* (2019) [16]	Health-EDRM / disaster research methods/ethics	Expert meeting report/thematic synthesis	Identify key methodological and ethical priorities for Health-EDRM and propose actions to strengthen research standards
Blanchet *et al.* (2017) [17]	Humanitarian crises (conflict, displacement, natural disasters)	Systematic review	Assess the quantity and quality of evidence on public health interventions in humanitarian crises and identify research gaps across health topics
Chou *et al.* (2021) [18]	Humanitarian emergencies; RMNCH program planning	Conceptual framework/methodological viewpoint	Describe the H-LiST approach, its rationale, implementation experiences, strengths, limitations, and future development needs
Christian *et al.* (2014) [19]	Pandemics and mass critical care emergencies	Consensus statement (modified Delphi); expert review	Identify and synthesise legal frameworks, obligations, and protections required for mass critical care planning and crisis standards of care
Devisch *et al.* (2017) [20]	Post-disaster suffering and long-term recovery	Conceptual/philosophical analysis with phenomenological framework	Explore ethical debriefing as a non-medical, narrative, care-based tool to support long-term recovery and existential suffering of disaster survivors
Eckenwiler *et al.* (2015) [21]	Disaster research ethics (various disaster and humanitarian settings)	Conceptual paper with case analyses	Propose the RTR model to strengthen ethical oversight through ongoing, adaptive, real-time responsiveness during disaster research
Hung *et al.* (2021) [22]	Health EDRM workforce development	Perspective/conceptual analysis	Identify evidence gaps and propose a multi-method project (literature reviews, case studies, Delphi) to produce recommendations for Health EDRM workforce development
Hunt *et al.* (2016) [23]	Disaster research ethics (LMIC contexts)	Qualitative study (interpretive description; 15 interviews with REC members)	Identify key elements (timeliness, responsiveness, rigour) shaping effective ethics review of disaster research
Jillson *et al.* (2019) [24]	Disaster response; evidence use; stakeholder engagement	Policy Delphi study (two rounds) and focus groups	Identify stakeholder perspectives on evidence use, barriers, ethical/legal/social issues, and ways to strengthen disaster response systems
Johnson and Vindrola-Padros (2017) [25]	Rapid qualitative research during complex health emergencies	Systematic review	Methods used, study timeframes, research team composition, benefits and limitations of rapid qualitative methods
Khorram-Manesh *et al.* (2024) [26]	Disasters and public health emergencies	Mixed methods (Delphi and survey with scenarios)	Explore the feasibility, challenges, and advantages of applying ethical approaches, including PCC, in disaster and emergency management
Koenig *et al.* (2017) [27]	Public health and disaster science	Conceptual commentary/viewpoint	Argue for replacing ‘lessons learned’ with evidence-based translational and implementation science to strengthen disaster medicine
Kohrt *et al.* (2019) [28]	Humanitarian crises (conflict, displacement, natural disasters, outbreaks)	Analysis/perspective paper	Identify evidence gaps and outline strategies to catalyse ethical, rigorous, actionable research in humanitarian crises
Leresche *et al.* (2020) [29]	Humanitarian settings, protracted crisis, Syrian refugee context	Conceptual analysis and reflective case study	Analyse a multidisciplinary research partnership (ICRC–MoPH–Harvard FXB) and identify factors enabling operational research in protracted conflict settings
Mena and Hilhorst (2022) [30]	Disaster research ethics in conflict settings	Qualitative reflective analysis of fieldwork experience; literature-informed conceptual paper	Everyday ethics; safety and security; equitable collaboration; participatory approaches; confidentiality; remote research ethics
Mezinska *et al.*., 2016 [31]	Disaster research ethics guidelines	Systematic qualitative review (constant comparative method)	Review and compare ethical guidelines for disaster research; identify core themes and gaps
Packenham *et al.*., 2017 [32]	Disaster research involving human subjects	Consensus working-group report	Develop 15 IRB recommendations addressing ethical, regulatory, and procedural challenges in reviewing disaster-related research
Pierce *et al.* (2017) [33]	Long-term care facilities in natural disasters (hurricanes, floods, earthquakes, pandemics)	Systematic/narrative literature review	Identify facility/disaster characteristics and interventions influencing preparedness and outcomes in LTCFs
Roy *et al.* (2011) [34]	Disasters in developing countries	Systematic review/evidence assessment	Assess the quantity, quality, themes, and evidence levels of disaster research focused on developing countries
Shalash *et al.* (2022) [35]	Humanitarian crises; health information systems	Analysis paper	Examine challenges and propose actions for standardised data collection, data sharing, and coordination among humanitarian agencies
Tansey *et al.* (2017) [36]	Natural disasters (LMIC settings)	Qualitative study (Interpretive Description; 15 interviews with REC members)	Identify how ethical concerns differ between disaster and non-disaster research from the perspective of REC members
Van Zandvoort *et al.* (2019) [37]	Humanitarian crises; infectious disease control	Review/conceptual analysis	Discuss barriers, rationale, and an evidence-generation pathway for PCC use in crises, including modelling, data needs, and operational strategies
Weine *et al.* (2021) [38]	Syrian refugee families: humanitarian emergency	Case study/applied implementation research	Describe challenges and lessons learned in developing and piloting a low-intensity transdiagnostic family support intervention in a humanitarian emergency setting
Wu *et al.* (2022) [39]	Cross-cultural challenges in hazards and disaster research	Technical note/framework development	Develop a definitional framework and training module to build cultural competence among disaster researchers

CBPR – community-based participatory research, CIOMS – Council for International Organizations of Medical Sciences, EDMR – eye movement desensitisation and reprocessing, ICRC – International Committee of the Red Cross, IRB – institutional review board, LMIC – low- and middle-income country, LTCF – long-term care facility,

MoPH – Ministry of Public Health, PCC – person-centred care, REC – research ethics committee, RMNCH – reproductive, maternal, newborn, and child health, RTR – real-time responsiveness

Contexts varied widely, from humanitarian crises and refugee displacement to pandemics, critical care emergencies and disaster research ethics, particularly in low- and middle-income country settings. Notably, many studies explicitly addressed research governance and ethics-review processes during emergencies, while others explored methodological shortcomings in evidence generation.

The aims of the studies reflected two dominant orientations: identifying and critiquing gaps in ethical or methodological practices and proposing frameworks, guidelines, or emergent methodological innovations to strengthen research conducted in disaster settings. This heterogeneity underscores the fragmented but evolving evidence base that shapes the scientific and ethical foundations of disaster medicine.

### Ethical and methodological themes identified across studies

The included studies revealed a broad constellation of ethical concerns, methodological constraints, and person-centred considerations relevant to research conducted in disaster settings (**Table 2**).

**Table 2:** Ethical and methodological themes identified across studies

Study, year, reference	Ethical issues identified	Methodological challenges	PCC/Patient-centred elements
Aarons (2018) [12]	Delays in ethics review; inequity; lack of coordination; autonomy of local RECs; vulnerable populations; need for transparent communication	Limited existing guidance; heterogeneity of REC structures; absence of common standards; small sample of informants; no empirical testing of proposed model	Some PCC elements via community representation; emphasis on communication, dignity, stakeholder engagement; respect for local context
Aarons (2019) [13]	Delays in ethics review; participant protection vs speed; equity; speculative pre-screening dilemmas; burden on RECs	Limited REC capacity in LMICs; impracticality of generic protocols; resource constraints; multi-jurisdiction complexity	Minimal explicit PCC; indirect emphasis on protecting participant welfare, dignity, and minimising burden on vulnerable communities
Abeysinghe and Leppold (2023) [14]	Competing framings of vulnerability; risk of exploitation; consent challenges; burdens on communities; researcher safety; over-research; inequities	Heterogeneous methods; lack of standard terminology; limited evidence; dominance of Western ethical models; difficulty capturing evolving contexts	Explicit emphasis on community engagement; participatory approaches; contextual consent; reflexivity; attention to dignity and social context
Afifi *et al.* (2020) [15]	Power imbalances; risk of tokenism; community mistrust; safety; confidentiality; positionality; structural oppression	Complex community dynamics; instability; lack of baseline data; mobility; challenges in sustaining partnerships; limited resources	Explicit PCC parallels: equitable partnerships, community voice, cultural humility, co-learning, respecting lived experience, long-term engagement
Aung *et al.* (2019) [16]	Ethical review delays; incentives misuse (*e.g.* food); stakeholder protection; need for universal ethical standards	Heterogeneous methodology; lack of standardised terms; difficulty translating findings across contexts; limited impact evaluation tools	Explicit emphasis on community participation; stakeholder involvement; respecting affected populations’ needs and voices
Blanchet *et al.* (2017) [17]	Equity in access; vulnerability; ethical limits of applying stable-setting evidence to crises; under-researched vulnerable groups; risks of poor-quality evidence guiding decisions	Methodological gaps; limited experimental designs; inconsistent reporting; absence of economic data; weak attribution; fragmented data systems	Indirect PCC: focus on needs of vulnerable populations; context sensitivity; identifying underserved groups; improving relevance of interventions to affected communities
Chou *et al.* (2021) [18]	Data gaps; inequities; limitations in intervention evidence; risks of misclassification; prioritisation challenges	Incomplete or low-quality humanitarian data; unstable populations; inconsistencies in indicators; limits of LiST assumptions; cost estimation constraints	Indirect PCC relevance through needs assessment, contextual tailoring, cross-sector collaboration, and prioritising interventions that improve population well-being
Christian *et al.* (2014) [19]	Duty to prepare; fairness; legality of triage; accountability; evacuation ethics	Limited evidence; reliance on expert opinion; heterogeneous legal contexts; real-time research limitations	Communication; transparency; maintaining dignity; expectations management
Devisch *et al.* (2017) [20]	Medicalisation of suffering; loss of meaning; existential distress; isolation; narrative silencing; dignity and autonomy concerns	Lack of empirical models; absence of standardised methods; subjective nature of suffering; difficulty operationalising phenomenology; limited evidence on long-term support	Explicit PCC orientation: narrative engagement, listening, dignity, meaning-making, individualised existential support, recognising lived experience
Eckenwiler *et al.* (2015) [21]	Vulnerability; exploitation risks; unpredictable ethical dilemmas; power asymmetries; confidentiality; justice; researcher moral distress	Lack of empirical guidance; rapidly changing contexts; inadequate REC capacity; unclear oversight processes; incomplete predictability of risks	Explicit PCC-related elements: attentiveness, responsiveness, dignity, community engagement, recognition of evolving needs
Hung *et al.* (2021) [22]	Inconsistent training standards; lack of competency frameworks; weak integration of community health workers; unclear roles; inequities; workforce shortages and unsafe conditions	Lack of global guidelines; fragmented programmes; weak governance; limited resources; insufficient evidence base; contextual variability	People-centred approach; capacity building; clarity of roles; standardised competencies; community engagement; resilience strengthening
Hunt *et al.* (2016) [23]	Vulnerability; re-traumatisation risk; political pressure; confusion between research and relief; participant protection vs urgency	Infrastructure disruption; lack of REC expertise; inconsistent procedures; need for rapid review; context gaps	Explicit PCC elements: sensitivity to context, minimising harm, respect for dignity; but PCC is not central as a formal framework
Jillson *et al.* (2019) [24]	Ethical, legal and social issues; resource allocation; consent difficulties; inequities; transparency; coordination failures	Limited evidence base; poor data sharing; inconsistent standards; communication barriers; fragmented stakeholders; political influences	Indirect PCC: community engagement, transparency, needs assessment, accessible communication, respect for affected populations
Johnson and Vindrola-Padros, (2017) [25]	Rapid data needs; trustworthiness; engagement with communities; researcher roles	Limited data quality; small sample sizes; bias; methodological gaps; missing timeframe details	Early community leader involvement; continuous sharing of findings; actionable recommendations with policymakers
Khorram-Manesh *et al.* (2024) [26]	Ethical dilemmas in triage; resource scarcity; balancing utilitarian and person-centred values; patient rights vs crisis constraints	Limited empirical evidence; scenario-based data; diverse international contexts; complex interaction of ethical frameworks	Explicit PCC focus; autonomy, values, preferences; individualised care when feasible; need for communication, dignity, flexible surge capacity
Koenig *et al.* (2017) [27]	Ethical concern over reliance on anecdotal ‘lessons’; risks of non-scientific decision-making; equity and population-level implications	Difficulty generating causal evidence; predominance of descriptive studies; unstable environments; lack of rigorous methodologies	Indirect PCC relevance: focus on population outcomes, evidence-based practice, structured communication, and knowledge transfer to improve the health of affected communities
Kohrt *et al.* (2019) [28]	Vulnerability; consent challenges; equity; risk–benefit dilemmas; political sensitivity; researcher safety; participant burden	Lack of baseline data; disrupted info systems; unstable settings; absence of control groups; limited funding; methodological inflexibility	Explicit PCC parallels: community engagement, cultural sensitivity, local leadership, addressing priority needs, improving well-being, respecting lived experience
Leresche *et al.* (2020) [29]	Power differentials; consent and confidentiality; vulnerability; risk of harm; ethical trade-offs in sampling, data use and community engagement	Methodological constraints; bias; lack of baseline data; GIS limitations; unstable funding; security/logistics; divergent analytic cultures	Explicit PCC parallels: trust-building, respect, community voice, safeguarding, equitable collaboration, meeting population needs
Mena and Hilhorst (2022) [30]	Everyday ethics; informed consent challenges; confidentiality risks; decolonising research; safety and security for participants and researchers	Lack of guidance for ethical dilemmas; inaccessible areas; limitations of remote research; power imbalances; inconsistent REC procedures	Continuous reflection; adaptive research processes; equitable collaboration; transparency; prioritising safety; responsible communication
Mezinska *et al.* (2016) [31]	Vulnerability; exploitation; re-traumatisation; unrealistic expectations; justice; confidentiality; dilemmas in REC review	Broad, inconsistent concepts; lack of standardised guidance; heterogeneity of guidelines; limited empirical evidence; unclear risk–benefit tools	Explicit emphasis on dignity, avoiding overburdening; community involvement; safeguarding vulnerable groups; informed consent challenges
Packenham *et al.* (2017) [32]	Vulnerability; unmet needs; coercion risks; consent confusion; confidentiality threats; participant burden; equity gaps; risks of re-traumatisation	Time pressures; insufficient IRB expertise; infrastructure disruption; lack of standardised context information; feasibility issues; inconsistent guidelines	PCC-parallels: identifying unmet needs; protecting dignity; transparent communication; participant-centred timing; community engagement; respecting capacity for consent
Pierce *et al.* (2017) [33]	Duty to protect vulnerable LTCF residents; ethical complexity of evacuation vs shelter-in-place; equity in resource allocation	Heterogeneous observational data; lack of high-level evidence; no RCT feasibility; inconsistent preparedness metrics	Limited explicit PCC; focus on vulnerability, dignity, continuity of care, psychological well-being
Roy *et al.* (2011) [34]	Publication bias; underrepresentation of vulnerable populations; ethical concerns in research on low-resource settings	Lack of high-quality evidence; predominance of level IV–V studies; weak methodology; limited data from developing regions	Minimal explicit PCC; implicit focus on equity and needs of vulnerable populations
Shalash *et al.* (2022) [35]	Inequities in aid distribution; risk of misinformed decisions; donor-driven agendas; local exclusion	Lack of standardised indicators; poor data quality; duplication of assessments; inconsistent coordination; missing baseline data	Limited PCC elements; indirect focus on local involvement, relevance of data, community-informed priority setting
Tansey *et al.* (2017) [36]	Justification; heightened vulnerability; re-traumatisation; confidentiality/safety risks; limits of community engagement	Unstable settings; evolving risks; lack of mechanisms for ongoing oversight; inconsistent REC practices; limited empirical guidance	PCC-related elements implicit: dignity, minimising harm, context sensitivity, tailored consent; emphasis on community engagement challenges
Van Zandvoort *et al.* (2019) [37]	Inequity in vaccine access; ethical dilemmas in prioritisation; uncertain benefits across ages; vulnerability of crisis-affected populations	Inadequate surveillance; absence of context-specific data; uncertain indirect effects; difficulty in implementing trials; logistic and cost constraints	Indirect PCC relevance via protecting high-risk groups; tailoring to local disease burden; community-relevant prioritisation; improving equitable access
Weine *et al.* (2021) [38]	Weak partnerships; unfamiliarity with task-sharing; limited cultural and language competence; gender-related barriers; stigma; refugee fears; extreme socioeconomic hardship	Lack of research capacity; limited local mental health infrastructure; difficulties in recruitment and retention; inconsistent collaboration structures	Explicit PCC elements: family-centred approach, cultural tailoring, community collaboration, respect for gender norms, responsiveness to lived experience
Wu *et al.* (2022) [39]	Power imbalances; trust and rapport challenges; risks of miscommunication; lack of cultural awareness can cause ethical harm	Rapid data collection conflicts; limited local engagement; lack of standardised cultural competence measures	Explicit PCC parallels: cultural humility, respect, reflexivity, community partnership, sensitivity to participant needs

GIS – Geographic Information System, IRB – institutional review board, LMIC – low- and middle-income country, LTCF – long-term care facility, PCC – person-centred care, RCT – randomised-controlled trial, REC – research ethics committee

### Ethical issues in disaster research

Ethical complexities were pervasive across the included studies and frequently centred on the tension between the urgency of conducting research during emergencies and the obligation to protect participants. Several authors described delays or inequities in ethics review processes, particularly in low-resource settings, where limited capacity and fragmented procedures jeopardise timeliness and fairness in decision-making [12,13,23]. Many studies emphasised the heightened vulnerability of participants in disaster and humanitarian contexts, underlining the risks of exploitation and re-traumatisation among conflict-affected, displaced, or otherwise marginalised populations [28,30,31]. Challenges related to informed consent were also prominent: authors highlighted difficulties in ensuring clarity and voluntariness when individuals are under acute stress, facing insecurity, or dependent on aid providers, raising concerns about coercion, undue influence, and therapeutic misconception [14,32,36].

Power asymmetries between international researchers, humanitarian organisations, and local communities were another recurring theme, often linked to mistrust, perceived tokenism, and structural forms of oppression [15,39]. In this context, several papers noted the moral distress experienced by researchers and ethics committee members who must make decisions under conditions of radical uncertainty and rapidly evolving risk–benefit profiles [21]. Equity-related concerns were also widely reported, including differential access to care, unequal distribution of resources, and the systematic exclusion of certain groups from research or services [17,37]. Across the literature, ethical issues rarely appeared in isolation; rather, they were deeply entangled with structural inequities, weak or overstretched governance systems, and the operational constraints that characterise disaster and humanitarian environments.

Importantly, several studies implicitly or explicitly framed ethical challenges as evolving across different temporal phases of crises, highlighting the need for person-centred approaches that extend beyond acute response. During the immediate response phase, ethical concerns focused on preserving dignity in triage communication, enabling rapid yet respectful elicitation of patient preferences, safeguarding vulnerable individuals, ensuring access to interpreters or cultural mediators, maintaining family contact, and upholding basic information rights. As crises transitioned into stabilisation phases, ethical attention shifted toward continuity of care, medication and chronic disease management, psychosocial support, navigation of fragmented services, and identification of unmet social needs. In protracted crises and recovery phases, ethical considerations increasingly encompassed rehabilitation, trauma-informed and long-term mental healthcare, reintegration into education or employment, sustained support for caregivers, access to legal and administrative assistance, and meaningful community participation in recovery processes.

Taken together, these findings suggest that ethical challenges in disaster research cannot be adequately addressed through episodic or crisis-limited interventions alone. Rather, the literature points toward the necessity of integrated, person-centred system approaches capable of supporting ethical practice across acute response, stabilisation, and long-term recovery, across crisis conditions and transitions to more stable health and social care systems.

### Methodological challenges

Methodological barriers were consistently identified across the studies and contributed substantially to the limitations of the existing evidence base in disaster medicine. Several authors pointed to the absence of rigorous or context-appropriate study designs, noting a persistent reliance on descriptive, observational, or anecdotal accounts in place of more robust analytic approaches [27,34]. Fragmented data systems, inconsistent indicators, and the paucity of reliable baseline information were repeatedly described as major obstacles to both internal and external validity [23,35]. In many settings, high-quality experimental designs such as randomised controlled trials were deemed infeasible or ethically problematic, particularly in the acute phase of disasters or outbreaks, where equipoise is unstable and conditions change rapidly [33].

Heterogeneity and lack of harmonisation in ethics review processes across jurisdictions further complicated multi-site and cross-border research, with authors describing challenges in coordinating requirements, timelines, and standards among different research ethics committees and regulatory bodies [12,13,23,32]. Rapidly changing field conditions and operational disruptions frequently undermined methodological consistency, impeded follow-up, and limited the feasibility of longitudinal or implementation research [21,25]. In addition, limited local research capacity, constrained resources, and fragile infrastructures, particularly in humanitarian crises and low- and middle-income countries, were noted as significant barriers to designing and sustaining rigorous studies [28,38]. Taken together, these challenges illustrate a persistent structural mismatch between conventional research methodologies and the realities of disaster contexts, underscoring the need for adaptive, flexible, and ethically grounded approaches to evidence generation.

### Person-centred and patient-centred elements

Although PCC/PCE did not constitute a dominant or consistently articulated framework across the included studies, many papers nonetheless engaged, explicitly or implicitly, with principles aligned with PCC. Some authors emphasised dignity, autonomy, and the lived experience of participants as central ethical commitments in disaster and post-disaster settings, emphasising the importance of narrative engagement, listening, and recognition of existential suffering [20,31]. Others highlighted stakeholder engagement and community participation as key strategies for mitigating power imbalances, strengthening trust, and enhancing the contextual relevance and acceptability of research [14,15].

Cultural humility and contextual sensitivity emerged as recurrent themes, particularly in cross-cultural, refugee, or migrant contexts, where researchers and practitioners must navigate complex social norms, language barriers, and histories of trauma or marginalisation [28,39]. Transparent communication and shared decision-making were also identified as important components of ethically robust research oversight, especially in relation to ethics review processes and communication with affected communities [12,13]. Several studies approached person-centredness indirectly, for example by focusing on the needs of vulnerable or underserved groups, the fairness of aid distribution, or the alignment of interventions with community-defined priorities [17,34]. Explicit reference to person-centred or patient-centred care as a formal methodological or ethical framework was uncommon, appearing in only a small minority (<25%) of the included studies [21,26]. More frequently, person-centred principles appeared indirectly through related concepts such as dignity, participation, autonomy, communication, continuity, cultural humility or responsiveness to lived experience, without being formally labelled as PCC/PCE. The apparent underrepresentation of PCC should therefore be interpreted cautiously, as it may reflect semantic variation and conceptual overlap rather than the complete absence of person-centred thinking.

Taken together, these findings highlight a fragmented but increasingly reflexive evidence base, in which ethical imperatives, methodological constraints, and emerging person-centred perspectives intersect to shape the future agenda for disaster medicine research.

## DISCUSSION

We synthesised ethical and methodological challenges in disaster health research, using PCC as an interpretive lens rather than as the primary object of analysis. We examined how existing research practices align with person-centred principles and how ethical commitments are operationalised within research design, thereby linking normative ethics with methodological implementation. With this scoping review, we demonstrate that the evidence base for disaster medicine remains conceptually developed yet methodologically constrained, with profound ethical tensions that frequently undermine the feasibility of traditional research designs and limit the operationalisation of PCC.

Across the 28 included studies, ethical concerns, particularly vulnerability, inequity, power asymmetries, and challenges to informed consent, emerged not as secondary issues but as structural determinants of what research is possible in crisis settings. At the same time, methodological barriers such as unstable environments, weak data systems, heterogeneous ethics review processes, and the impracticality of experimental designs continue to constrain robust evidence generation. Although elements of person-centred and patient-centred care appeared throughout the literature, they were rarely formalised, and only a small subset of studies explicitly framed PCC/PCE as an essential methodological or ethical requirement. Together, these findings show a maturing yet incomplete field at a critical inflexion point, where ethical imperatives, adaptive methodologies, and person-centred principles must converge to shape a more coherent and globally relevant research paradigm for disaster contexts. Notably, the identification of these domains was influenced in part by the conceptual framing of the review and the design of the search strategy, which explicitly targeted ethics, methodology, and PCC. While themes were developed inductively from the included studies, the analytic structure therefore reflects a combination of emergent patterns and conceptually guided categorisation.

These findings should also be interpreted in the context of existing international guidance on disaster and humanitarian research ethics. Several frameworks have been developed to support ethical research conduct in crisis settings, including the Council for International Organizations of Medical Sciences Guideline 20 on research in disasters [40], the Nuffield Council on Bioethics guidance on humanitarian research ethics [41], and operational ethics frameworks developed by organisations such as Médecins Sans Frontières and the Research for Health in Humanitarian Crises programme [42]. Previous analyses have highlighted the complexity of conducting ethically robust research in humanitarian crises, particularly regarding issues of vulnerability, informed consent, community engagement, and power asymmetries [43]. Taken together, these frameworks reinforce the argument that ethical and methodological considerations in disaster research must be addressed through context-sensitive and adaptive approaches. An additional consideration is the geographic and institutional distribution of the evidence base. Many of the included studies originate from high-income country institutions or global policy networks, while the burden of disasters disproportionately affects low- and middle-income settings. This imbalance raises important questions about whose perspectives shape the conceptualisation of disaster research ethics and methodology. It also reflects broader structural asymmetries in knowledge production, where local voices and context-specific experiences may be underrepresented. Addressing these gaps is essential for advancing more equitable and contextually grounded approaches to disaster health research.

The patterns identified across the included studies highlight a persistent structural misalignment between the ethical demands of disaster research and the methodological tools currently available. Therefore, it is important to distinguish between ethical issues related to clinical care delivery and those specific to research conduct. While some studies discuss triage, communication, evacuation, or bedside decision-making in clinical contexts, these elements are considered here only insofar as they shape the ethical environment in which disaster research is designed, reviewed, or conducted, rather than as a comprehensive treatment of disaster clinical ethics. Ethical concerns, particularly those relating to vulnerability, coercion, and re-traumatisation, were most prominent in studies focusing on conflict-affected or displaced populations, where the risks associated with participation are amplified by insecurity and dependency [28,30,31]. Authors repeatedly emphasised that informed consent in such contexts is not a procedural formality but an ethically contested process shaped by fear, urgency, and asymmetric power relationships [14,32,37]. Even in settings with stronger governance, delays or inequities in ethics review were seen as barriers to timely and context-appropriate research, particularly in lower-resource countries where research ethics committee capacity is limited [12,13,23]. Methodologically, the field remains constrained by the dominance of descriptive and observational designs. Several studies noted that the instability of disaster environments, the absence of baseline data, and rapidly shifting priorities make rigorous prospective research extremely challenging to execute [27,34]. Fragmented information systems and inconsistent indicators were widely recognised as structural impediments to both internal validity and cross-context comparability, thereby limiting the development of generalisable knowledge [22,35]. Meanwhile, the feasibility and ethics of experimental approaches, such as randomised trials, continue to be questioned, given the fluidity of risk-benefit profiles and the moral unease surrounding allocation decisions during crises [33].

Despite these limitations, several papers proposed adaptive methodological strategies that better reflect the realities of crisis research. Rapid qualitative methods were highlighted for their ability to generate actionable insights under severe time constraints [25], while models such as real-time responsiveness were suggested as mechanisms for strengthening continuous ethical oversight in volatile environments [21]. Other authors called for enhanced collaboration between international actors, local health authorities, and affected communities as a means of improving both methodological feasibility and ethical integrity [29]. The treatment of person-centred and patient-centred care across the literature underscores a critical gap in the current evidence base. While several studies engaged with PCC principles, such as dignity, autonomy, narrative engagement, and cultural humility, these elements were rarely formalised within explicit methodological frameworks [20,28,39]. Only a small subset explicitly positioned PCC/PCE as a guiding approach to disaster research design or emergency care decision-making [21,26]. Notably, the literature often operationalised person-centred principles implicitly, for example through community participation, protection of vulnerable groups, or attention to communication and trust, rather than through explicit use of PCC terminology. This suggests that the apparent underrepresentation of PCC may partly reflect semantic variation rather than complete conceptual absence. Nevertheless, the lack of explicit articulation limits the ability to systematically integrate and evaluate person-centred approaches within disaster research, highlighting an important area for further methodological development. Collectively, these findings suggest that advancing disaster medicine requires more than refining existing methods; it requires reframing the methodological and ethical foundations of the field. The convergence of ethical imperatives, resource constraints, evolving global threats, and an emerging recognition of person-centred principles offers a strategic moment to redefine what counts as rigorous, ethical, and contextually meaningful research in disaster settings.

The following recommendations should be interpreted primarily as forward-looking implications for future disaster health research and research governance rather than as direct evidence-based or clinical guidelines. They are informed by patterns identified in the included studies but extend beyond the immediate evidentiary base of the review to highlight potential directions for conceptual and methodological development. The synthesis of methodological, ethical, and person-centred challenges across the included studies reveals a field that is approaching a moment of significant transformation. To progress beyond a fragmented and predominantly descriptive evidence base, disaster medicine must adopt research strategies that remain ethically robust while also being operationally feasible in unstable, resource-constrained environments. The patterns identified in this review suggest several interlinked directions that can help establish a more coherent and globally relevant research agenda.

One possible future direction, suggested indirectly by the reviewed literature on fragmented data systems, communication barriers, and ethical decision-making, is the development of digital and person-centred decision-support tools tailored to disaster settings, designed to integrate clinical care with broader system-level considerations, including social needs, continuity planning, secure cross-sector data sharing, and communication supports responsive to linguistic and accessibility requirements. Across the literature, investigators consistently highlighted fragmented data systems, inconsistent indicators, and the lack of mechanisms for integrating patient perspectives into clinical or operational decision-making, problems documented in studies by Shalash *et al.* [35], Hung *et al.* [22], Afifi *et al.* [15], and Wu *et al.* [39]. At the same time, ethical analyses drew attention to recurring concerns related to dignity, autonomy, cultural context, and trust, principles closely aligned with PCC but rarely operationalised within disaster environments, as shown by Devisch *et al.* [20], Mezinska *et al.* [31], and Kohrt *et al.* [28]. The creation of interoperable platforms capable of integrating clinical and contextual information, patient values, communication needs, and real-time operational data would directly address these problems by embedding person-centred ethical parameters into high-stakes decisions such as triage, evacuation, and resource allocation. Such systems would go beyond existing tools by explicitly incorporating ethical considerations that reduce moral distress among responders and support ethically defensible decision-making, as emphasised by Eckenwiler *et al.* [21] and by Khorram-Manesh *et al.* [26]. This direction aligns well with ongoing developments in global digital health and could be piloted within the World Health Organization, Horizon Europe, or EU Civil Protection initiatives.

A second possible direction for future development is the establishment of structured training frameworks that more explicitly embed person-centred and ethical decision-making within disaster response. Such frameworks should support cross-sector training across healthcare, social care, and humanitarian organisations, with a focus on managing moral distress, applying trauma-informed approaches, and sustaining effective communication under conditions of scarcity. Anchored in person-centred principles at micro, meso, and macro levels, these training initiatives can strengthen continuity of care and cross-sector coordination across all phases of crisis response. Although elements of PCC appear sporadically across the literature, they remain largely absent from formal disaster preparedness curricula, which tend to prioritise logistical efficiency and surge capacity over relational and ethical competencies. Studies by Afifi *et al.* [15], Abeysinghe and Leppold *et al.* [14] and Christian *et al.* [19] illustrate how communication, equity, vulnerability, and community engagement often determine the acceptability and impact of interventions. Training programmes should therefore incorporate competencies related to ethical decision-making under resource scarcity, communication with distressed or marginalised populations, the management of moral distress among responders, as highlighted by Eckenwiler *et al.* [21], and the cultural and contextual sensitivities emphasised by Wu *et al.* [39]. Simulation-based learning, including advanced virtual-reality scenarios and structured ethical case discussions, provides a promising modality for preparing responders to navigate the tensions between utilitarian imperatives and person-centred commitments. Such programmes may enhance ethical preparedness, build trust, and improve outcomes for affected populations.

A third possible area for methodological development is the creation and standardisation of quality indicators capable of evaluating person-centred dimensions of disaster response and recovery. Such indicators should capture system-level resilience and continuity following displacement, documentation of individual preferences and communication needs, timeliness of linkage to social support, equity of access for vulnerable groups, and patient-reported experiences of dignity and respect during triage and acute care [40]. Indicators should also reflect cross-sector coordination across care transitions and enable follow-up assessment over extended recovery periods.

One of the clearest gaps that emerged is the absence of measurable criteria for assessing whether disaster responses uphold dignity, autonomy, equity, and continuity of care. Although several studies discussed community engagement, vulnerability, or the needs of underserved groups, none offered systematic frameworks for evaluating person-centred performance in emergencies, a gap evident in the work of Blanchet *et al.* [17], Roy *et al.* [34], and Tansey *et al.* [36]. Therefore, there is a strong rationale for creating dynamic indicators suited to emergency conditions, indicators that assess, for example, the timeliness and clarity of communication during triage, the appropriateness of decision support for diverse cultural contexts, the extent of shared decision-making possible under constraints, the continuity of care throughout surge phases, and the degree to which vulnerable groups experience equitable access. Integrating such indicators into existing benchmarking systems, including the World Health Organization quality-of-care frameworks, Organisation for Economic Co-operation and Development health metrics, and EU preparedness assessments, would enable meaningful comparison across settings and strengthen accountability in disaster response [44]. These proposals should be read as hypothesis-generating and agenda-setting implications derived from thematic gaps in the literature, rather than as direct evidence-based recommendations validated by comparative intervention studies.

Taken together, these recommendations offer a forward-looking roadmap that builds directly on our findings. They respond to the ethical imperatives identified across the literature, including concerns about vulnerability, informed consent, and inequity. We address the methodological barriers highlighted by numerous authors, including weak data systems, limited research capacity, and the infeasibility of conventional study designs. We also confront the underdeveloped integration of person-centred principles, which, despite their ethical prominence, remain insufficiently embedded in disaster research and practice. By advancing these directions, disaster medicine can evolve into a field in which evidence generation, operational decision-making, and ethical integrity reinforce rather than constrain one another.

### Strengths and limitations

We offer one of the most comprehensive attempts to date to map the methodological, ethical, and person-centred dimensions of research conducted in disaster settings. A major strength of the review is our systematic and transparently documented methodology, developed *a priori* and registered on the Open Science Framework. We followed PRISMA-ScR guidance, applied rigorous dual-reviewer screening and extraction procedures, and used an inductive thematic synthesis that allowed the identification of cross-cutting patterns not visible in single-study analyses. The structured tabular mapping of study characteristics and analytic dimensions consolidated highly dispersed evidence base and provides the first integrated overview linking ethical challenges, methodological constraints, and the limited but emerging presence of PCC in disaster medicine. With this review, we establish a conceptual foundation on which future empirical and methodological innovation can build. Another strength lies in the breadth of included material. By synthesising conceptual analyses, qualitative studies, systematic reviews, consensus statements, and reflective papers across a wide array of crisis contexts, we captured the heterogeneity that defines disaster medicine as a field. This diversity allowed us to identify thematic convergence across settings, particularly around vulnerability, inequity, weak data systems, and the difficulty of conducting ethically robust research under unstable conditions, which strengthens the global relevance of the findings. Importantly, we highlighted that despite growing international emphasis on dignity, autonomy, and shared decision-making, explicit operationalisation of PCC remains exceptionally rare in disaster-related research, underscoring a gap that has not been systematically described before.

At the same time, the review has several limitations that reflect broader constraints in the underlying literature. First, the available evidence on PCC in disaster settings is extremely limited and conceptually underdeveloped. Only a small subset of studies engaged directly with PCC principles, and even fewer articulated methodological frameworks that could meaningfully integrate dignity, autonomy, or lived experience into research design. As a result, our conclusions regarding PCC-related gaps rely in part on inference from ethical, cultural, or participatory themes rather than on explicit PCC scholarship. However, this highlights the very gap the review aims to expose: the absence of structured, patient-centred thinking in a field where it is ethically indispensable. Second, although the search strategy was comprehensive and included three major databases, the fragmented nature of disaster medicine research means that relevant insights may exist in grey literature, humanitarian agency reports, or operational documents not captured in peer-reviewed sources. Some of these materials may contain practical methodological innovations or ethical deliberations that were beyond our scope. Nonetheless, limiting the search to peer-reviewed publications ensured methodological consistency and alignment with our objective of defining the scientific evidence base. Third, the scoping review design does not permit formal appraisal of study quality or the determination of causal relationships. Given the predominance of descriptive and conceptual work, this limitation is inherent to the state of the literature. Rather than weakening the review, it reinforces the conclusion that rigorous, context-appropriate methodologies remain underdeveloped and urgently needed. Finally, heterogeneity across study types, crisis contexts, and ethical frameworks limited the ability to generalise findings to specific disaster settings. However, our goal was not to derive uniform prescriptions but to illuminate structural patterns across diverse environments. The consistency with which core themes, vulnerability, inequity, methodological fragility, and the marginalisation of person-centred values, appeared across contexts suggests that the identified gaps are not incidental but foundational to current research practice in disaster medicine.

Overall, the strengths of our study lie in its systematic approach, conceptual clarity, and ability to synthesise a highly fragmented evidence base. Its limitations, many of which stem from the scarcity and unevenness of existing scholarship, underscore the urgency of developing ethically grounded, methodologically adaptive, and person-centred research paradigms for disaster settings. Additionally, the geographic and institutional distribution of the included literature may reflect broader imbalances in global knowledge production, with a predominance of studies originating from high-income settings and potential underrepresentation of perspectives from low- and middle-income contexts.

## CONCLUSIONS

The evidence base for disaster medicine appears conceptually rich but structurally fragile. Across this heterogeneous body of work, ethical and methodological constraints point to a persistent gap in the explicit operationalisation and evaluation of PCC within disaster research, although related principles may often be present under adjacent terminology. Rather than offering definitive conclusions, we offer an interpretive synthesis of a heterogeneous, evolving, and conceptually diverse field, highlighting ethical imperatives and methodological adaptation. Future progress will likely depend on adaptive research approaches, context-responsive ethical oversight, and more explicit integration of person-centred considerations into study design and evaluation, particularly in protracted crisis and recovery settings
